# Advancing endodontic imaging: a high-resolution comparison between CBCT and micro-CT in the danger zone

**DOI:** 10.2340/biid.v13.46618

**Published:** 2026-08-05

**Authors:** Gabriel de Toledo Telles-Araujo, Raimundo Sales de Oliveira Neto, Mariana Quirino Silveira Soares, Heitor Marques Honório, Viviane Almeida Sarmento, Marco Antônio Hungaro Duarte, Izabel Regina Fischer Rubira-Bullen

**Affiliations:** a School of Medicine, Federal University of Bahia, Salvador, Brazil; b Department of Operative Dentistry, Endodontics, and Dental Materials, Bauru School of Dentistry, University of São Paulo, Bauru, Brazil; c Oral Radiology Division, São Leopoldo Mandic Research Institute, Campinas, Brazil; d Department of Pediatric Dentistry, Orthodontics and Public Health, Bauru School of Dentistry, University of São Paulo, Bauru, Brazil; e School of Dentistry, Federal University of Bahia, State University of Feira de Santana, Bahia, Brazil; f Department of Surgery, Stomatology, Pathology and Radiology, Bauru School of Dentistry, University of São Paulo, Bauru, Brazil

**Keywords:** Cone-beam computed tomography, danger zone, endodontics, micro-computed tomography, root canal

## Abstract

**Objective:**

This study evaluated the accuracy of cone-beam computed tomography (CBCT) protocols for measuring submillimetric dentin thickness in the danger zone (DZ) of mandibular molars, using micro-computed tomography (micro-CT) as the reference standard.

**Materials and methods:**

Fifty-one extracted mandibular molars were scanned using micro-CT and two CBCT devices under different acquisition protocols (Standard, High Fidelity, High Resolution [HIRE], and Imaging System [i-CAT]). After three-dimensional image registration, dentin thickness in the DZ, located 2 mm below the furcation, was measured in both mesial canals.

**Results:**

All CBCT protocols significantly underestimated dentin thickness compared with micro-CT (*p* < 0.05), with discrepancies ranging from 4.5 to 8.6%. Underestimation was more pronounced in the mesiolingual canal. The HIRE protocol (0.125 mm voxel size) showed the smallest discrepancy, whereas the i-CAT protocol showed the largest.

**Conclusion(s):**

CBCT systematically underestimates dentin thickness in the DZ and is not metrically equivalent to micro-CT for submillimetric measurements. Measurement accuracy is influenced by acquisition parameters and cannot be explained by voxel size alone.

## Introduction

Micro-computed tomography (micro-CT) serves as the gold standard in ex vivo endodontic research, enabling highly accurate anatomical assessments [1–4]. However, its lack of clinical applicability has prompted the use of cone-beam computed tomography (CBCT) as a clinical alternative, necessitating validation studies against the micro-CT benchmark [1, 3, 5–8]. While the general utility of CBCT in endodontics is well established [9–11], recent technological advancements – such as smaller voxel sizes and higher spatial resolution – have enabled the visualization of submillimetric structures. Consequently, research has increasingly focused on evaluating CBCT’s accuracy for these detailed measurements, where image acquisition protocols are critical determinants of reliability [1, 10–13].

The complex anatomy of mandibular molars presents a considerable challenge in endodontic treatment. These teeth generally feature two roots: a mesial root that is flattened mesiodistally and widened buccolingually, and a typically straighter distal root containing one or two root canals [1, 14, 15]. A critical area of concern is the danger zone (DZ), the thin distal dentin wall of the mesial root adjacent to the furcation. This region’s inherently reduced dentin thickness renders it highly susceptible to iatrogenic complications like root perforation and vertical fractures during mechanized instrumentation [16].

Although interest in comparing dentin thickness between micro-CT and CBCT is growing, such studies demand rigorous methodologies. While micro-CT remains the gold standard for endodontic morphological assessment due to its superior resolution (19–41 μm) and ability to accurately define anatomical complexities such as lateral canals and apical deltas, its exclusive ex vivo application limits its direct clinical utility [17]. In contrast, CBCT offers a clinically viable, non-invasive alternative for in vivo assessment, demonstrating a strong correlation with micro-CT for dentin thickness measurement (intraclass correlation coefficient [ICC] = 0.988) [5].

However, a critical and clinically significant knowledge gap persists: all CBCT protocols, irrespective of field of view (FOV) or voxel size, systematically overestimate dentin thickness in the DZ compared to micro-CT, creating a false sense of security during root canal instrumentation [18]. The existing literature has documented this overestimation but has not yet provided validated correction factors or calibration methods to compensate for this systematic error. Furthermore, there is a notable absence of standardized measurement protocols, evidenced by a lack of consensus on specific anatomical landmarks and methodologies for evaluating the DZ [18].

To address these limitations, this study aims to evaluate measurement discrepancies of the mandibular molar DZ between micro-CT and various CBCT protocols, assessing their reliability for endodontic applications. Specifically, by establishing a correction model and a standardized measurement protocol, this research aims to translate the accuracy of micro-CT reference data into a clinically applicable framework, thereby enhancing the safety and reliability of CBCT-guided endodontic procedures.

## Materials and methods

**Figure 1:**
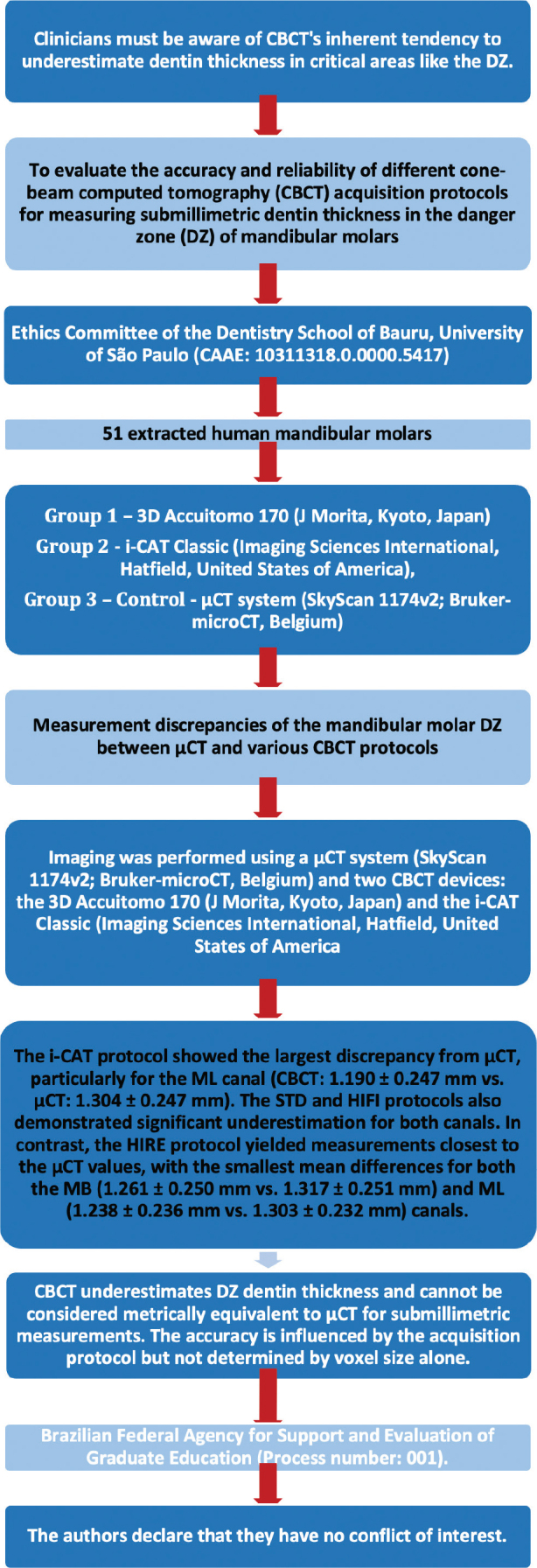
PRILE 2021 flowchart. CBCT: cone-beam computed tomography; micro-CT: micro-computed tomography; STD: Standard; HIFI: High Fidelity; HIRE: High Resolution; i-CAT: Imaging System; DZ: danger zone; MB: mesiobuccal; ML: mesiolingual.

The manuscript of this laboratory study has been written according to Preferred Reporting Items for Laboratory studies in Endodontology (PRILE) 2021 guidelines – Figure 1 [19].

### Sample selection

This study was approved by the local ethics committee. A power analysis was conducted a priori using G*Power software (v. 3.1 for Mac) with data from a previous study [1]. For a Wilcoxon/Mann-Whitney test, a sample size of 51 achieved 80% power (β = 0.20) to detect an effect size of 0.67 with an alpha level of 0.05. The Mann-Whitney test was selected for the sample size calculation as it provides a conservative estimate that does not rely on assumptions about the underlying data distribution. A sample of 51 extracted human mandibular molars was selected from a Brazilian population. Teeth were extracted due to periodontal disease, caries, or coronal fractures, and those with root fractures, resorption, calcifications, incomplete rhizogenesis, or prior endodontic treatment were excluded. Patient age and sex were not recorded.

### CBCT and micro-CT scanning

All teeth were coated with utility wax (Tenatex Red, Associated Dental Products Ltd, Kemdent Works, United Kingdom) to simulate the periodontal ligament space and mounted in the prepared alveoli of dry human jaws. The jaws themselves were externally coated with three layers of wax to mimic soft tissue. Imaging was performed using a micro-CT system (SkyScan 1174v2; Bruker-microCT, Belgium) and two CBCT devices: the 3D Accuitomo 170 (J Morita, Kyoto, Japan) and the Imaging System (i-CAT) Classic (Imaging Sciences International, Hatfield, United States of America). The detailed acquisition parameters for all scanners are provided in Table 1.

**Table 1:** CBCT and mCT acquisition protocols.

Protocol	Voxel	FOV	mA	kV	Exposure time	Dose
STD	200 μm	10x5cm	60mA	80kV	17,5s	901 mGy·cm²
HIFI	200 μm	10x5cm	60mA	80kV	30,8s	1580 mGy·cm²
HIRE	125 μm	6x6cm	60mA	80kV	30,8s	1320 mGy·cm²
i-CAT	200 μm	6x16cm	36.12mA	120kV	40s	NR
micro-CT	22.9 μm	NR	800mA	50kV	NR	NR

STD: Standard; HIFI: High Fidelity; HIRE: High Resolution; i-CAT: Imaging System; micro-CT: micro-computed tomography; FOV: field of view; mA: Milliamperage; kV: Kilovoltage; s: seconds; μm: micrometer; mGy·cm²: Milligray-square centimeter; NR: Not Reported; CBCT: cone-beam computed tomography.

### Sample analysis

All images were analyzed in a darkened room on a dedicated workstation with a high-resolution monitor (EIZO FlexScan S2000, 1600 × 1200 pixels; EIZO NANAO Corporation, Hakusan, Japan).

The CBCT scans were exported in Digital Imaging and Communications in Medicine (DICOM) format. A Volume of Interest (VOI) was selected, and three-dimensional intermodal registration between the CBCT and micro-CT datasets was performed using DataViewer software (Bruker-microCT). This registration involved manual alignment based on anatomical landmarks to ensure optimal spatial correspondence and minimize errors. To verify the accuracy of the three-dimensional registration procedure, the alignment was visually inspected in all three orthogonal planes (axial, sagittal, and coronal) for each specimen. The registered images were then exported in bitmap (BMP) format for subsequent measurement.

**Figure 2:**
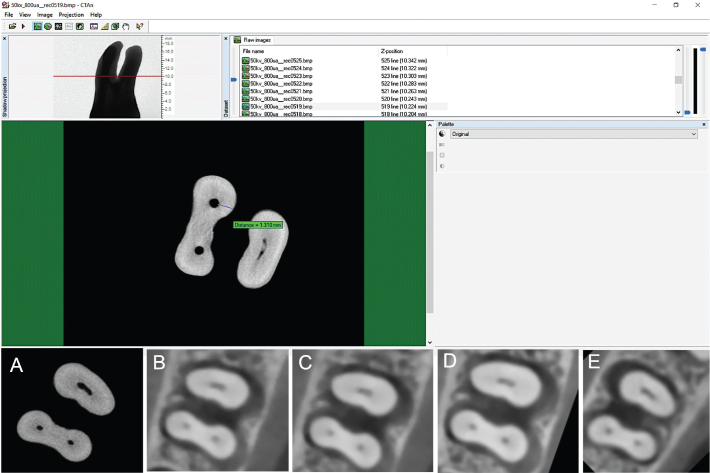
CTAn (v.1.12, Bruker-microCT) and visualization of the software interface, showing measurements in the DZ. Bottom: CBCT protocols (A) micro-CT; (B) STD; (C) HIFI; (D) HIRE; (E) i-CAT. CBCT: cone-beam computed tomography; micro-CT: micro-computed tomography; STD: Standard; HIFI: High Fidelity; HIRE: High Resolution; i-CAT: Imaging System; DZ: danger zone.

Linear measurements of the DZ were performed 2 mm below the bifurcation using CT-Analyser software (v.1.12, Bruker-microCT). On the axial slice, a line was drawn connecting the mesiobuccal (MB) and mesiolingual (ML) canals. A second line was drawn from the midpoint of this first line, perpendicularly towards the furcation, to measure the dentin thickness (Figure 2). This procedure was repeated for all CBCT acquisition protocols (Standard [STD], High Fidelity [HIFI], High Resolution [HIRE], and i-CAT), with the micro-CT measurements serving as the reference standard.

### Statistical analysis

Intraobserver agreement was evaluated using the ICC. For this purpose, a random subset comprising 20% of the sample was re-evaluated by the same examiner after a 30-day interval under identical conditions. The data were analyzed using SPSS (v.15.0, IBM, New York, United States of America) and Statistica software (v.12, TIBCO Software Inc., Palo Alto, CA, United States of America). The agreement between micro-CT and the different CBCT protocols was assessed using Bland-Altman analysis and linear regression. The final analysis employed paired *t*-tests after confirming normality of the paired differences (Shapiro-Wilk test, *p* > 0.05).

## Results

**Figure 3:**
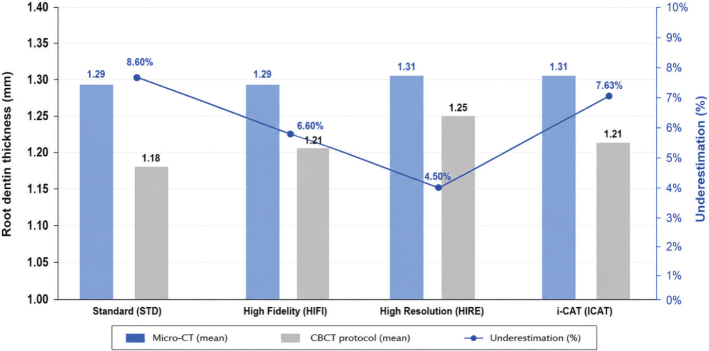
Bars represent the mean root dentin thickness obtained from the pooled measurements of the MB and ML canals at the three evaluated levels. Because these values correspond to pooled means derived from multiple anatomical locations, variability measures (SD or 95% confidence intervals) are presented in Tables 2–5 rather than in this summary figure. MB: mesiobuccal; ML: mesiolingual; CBCT: cone-beam computed tomography; micro-CT: Microcomputed Tomography; STD: Standard; HIFI: High Fidelity; HIRE: High Resolution; i-CAT: Imaging System.

All CBCT protocols consistently underestimated dentin thickness in the DZ compared to the micro-CT reference. When analyzed by canal, the ML canal exhibited greater underestimation than the MB canal across all protocols. The degree of underestimation varied by protocol. The i-CAT protocol showed the largest discrepancy from micro-CT, particularly for the ML canal (CBCT: 1.190 ± 0.247 mm vs. micro-CT: 1.304 ± 0.247 mm). The STD and HIFI protocols also demonstrated significant underestimation for both canals. In contrast, the HIRE protocol yielded measurements closest to the micro-CT values, with the smallest mean differences for both the MB (1.261 ± 0.250 mm vs. 1.317 ± 0.251 mm) and ML (1.238 ± 0.236 mm vs. 1.303 ± 0.232 mm) canals. Considering the mean values of the dentin thickness of the different protocols for the acquisition of the CBCT in comparison with the values obtained in micro-CT, a submillimetric underestimation of the values was observed in all protocols, with the HIRE protocol being the least underestimated (4.5%). The other values are shown in Figure 3.

A summary of the overall root dentin thickness measurements, including the mean values and the corresponding mean differences between micro-CT and each CBCT protocol, is presented in Tables 2–5. Detailed results are also provided according to canal (MB and ML) and anatomical measurement site (furcation, 2 mm, and 3 mm apical to the furcation). Overall, all CBCT protocols significantly underestimated root dentin thickness compared with the micro-CT reference (all *p* < 0.001).

**Table 2:** Mean(mm), standard deviation, and *p*-value/significance (mm) comparing micro-CT and STD

Root canal	Site	micro-CT		STD		*P*
		Mean	SD	Mean	SD	
**Mesiobuccal**	Bifurcation	1.400	0.250	1.293	0.239	**< 0.001**
	2 mm	1.275	0.244	1.198	0.242	**< 0.001**
	3 mm	1.234	0.237	1.112	0.252	**< 0.001**
**Mesiolingual**	Bifurcation	1.387	0.215	1.272	0.224	**< 0.001**
	2 mm	1.296	0.248	1.153	0.274	**< 0.001**
	3 mm	1.201	0.232	1.091	0.246	**< 0.001**
Mean difference (mm)		1.29		1.18		0.11

Values in bold: *p* < 0.05.

**Table 3:** Mean(mm), standard deviation, and p-value/significance (mm) comparing micro-CT and HIFI.

Root canal	Site	micro-CT		HIFI		*P*
		Mean	SD	Mean	SD	
**Mesiobuccal**	Bifurcation	1.400	0.250	1.307	0.235	**< 0.001**
	2 mm	1.275	0.244	1.210	0.251	**< 0.001**
	3 mm	1.234	0.237	1.155	0.253	**< 0.001**
**Mesiolingual**	Bifurcation	1.387	0.215	1.290	0.204	**< 0.001**
	2 mm	1.296	0.248	1.195	0.242	**< 0.001**
	3 mm	1.201	0.232	1.118	0.253	**< 0.001**
Mean difference (mm)		1.29		1.21		**0.08**

HIFI: High Fidelity.

Values in bold: *p* < 0.05.

**Table 4:** Mean(mm), mean difference (mm) standard deviation, and p-value/significance (mm) comparing micro-CT and HIRE. accuracy (Acc).

Root canal	Site	micro-CT		HIRE		*P*
		Mean	SD	Mean	SD	
**Mesiobuccal**	Bifurcation	1.418	0.264	1.360	0.248	**< 0.001**
	2 mm	1.291	0.252	1.249	0.245	**< 0.001**
	3 mm	1.244	0.239	1.176	0.257	**< 0.001**
**Mesiolingual**	Bifurcation	1.396	0.216	1.326	0.225	**< 0.001**
	2 mm	1.304	0.247	1.240	0.240	**< 0.001**
	3 mm	1.209	0.235	1.149	0.244	**< 0.001**
Mean difference (mm)		1.31		1.25		**0.06**

HIRE: High Resolution.

Values in bold: *p* < 0.05.

**Table 5:** Mean(mm), mean difference (mm) standard deviation, and p-value/significance (mm) comparing micro-CT and ICAT.

Root canal	Site	micro-CT		ICAT		*P*
		Mean	SD	Mean	SD	
**Mesiobuccal**	Bifurcation	1.418	0.264	13.29	0.271	**<0.001**
	2 mm	1.291	0.252	1.236	0.259	**<0.001**
	3 mm	1.244	0.239	1.177	0.251	**<0.001**
**Mesiolingual**	Bifurcation	1.396	0.216	1.266	0.239	**<0.001**
	2 mm	1.304	0.247	1.172	0.246	**<0.001**
	3 mm	1.209	0.235	1.134	0.257	**<0.001**
Mean difference (mm)		1.31		1.21		**0.10**

Values in bold: *p* < 0.05.

**Figure 4:**
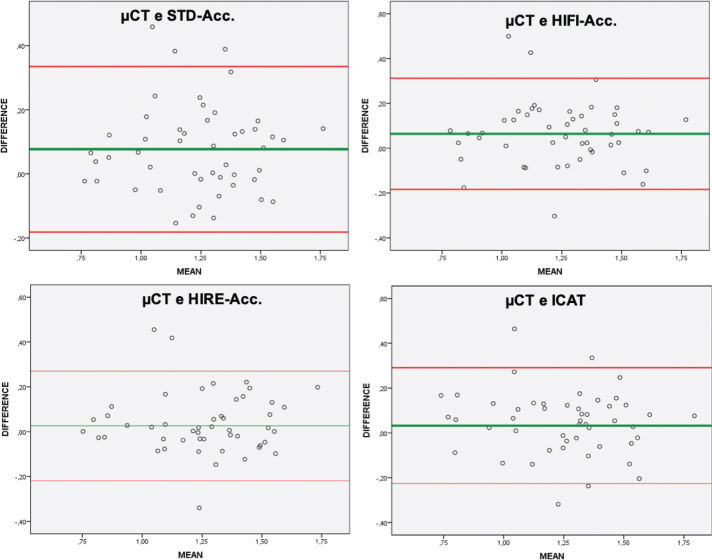
Bland-Altman plots with measurements of root dentin thickness between micro-CT and CBCT images. The green line represents the mean of the differences, and the limits of agreement are indicated by horizontal red lines. The mean difference was significantly different from zero, indicating a systematic bias between the methods and suggesting limited agreement, rather than interchangeability. CBCT: cone-beam computed tomography; micro-CT: micro-computed tomography; STD: Standard; HIFI: High Fidelity; HIRE: High Resolution; i-CAT: Imaging System.

The degree of agreement of measurements calculated by the Bland-Altman method showed that points of the present sample are located within the upper and lower (95%) confidence limits (mean standard deviation of 1.96). However, the differences were homogeneously distributed around an average value distant from zero, confirming that there was no agreement between CBCT and micro-CT (Figure 4). Likewise, from the linear regression test, it was concluded that there is no proportion bias between the gold standard (micro-CT) and the different protocols of the CBCT.

## Discussion

This study evaluated the accuracy of various CBCT acquisition protocols for linear measurements against the micro-CT gold standard. All CBCT protocols underestimated dentin thickness, with discrepancies ranging from 4.5 to 8.6%. This degree of underestimation could be clinically significant in cases where the DZ is naturally thin prior to intervention. The STD protocol showed the highest underestimation (8.6%), a finding consistent with previous studies reporting similar slight underestimations of CBCT compared to µCT [5, 6, 20].

While the paired *t*-test detected statistically significant differences between CBCT and micro-CT measurements (*p* < 0.05), this statistical significance does not automatically imply clinical relevance. The observed discrepancies ranged from 4.5 to 8.6%, which may appear modest in absolute terms. However, when applied to the clinical context of endodontic instrumentation, even these small differences can be meaningful, particularly in cases where the preoperative dentin thickness approaches critical thresholds. Preoperative assessment of dentin thickness in the DZ is critical for planning conservative instrumentation and avoiding iatrogenic complications. The literature recommends limiting instrumentation to a maximum of 0.5 mm and maintaining a minimum residual dentin thickness of 0.2–0.3 mm to prevent perforations and vertical root fractures [21, 22]. The underestimation observed in our CBCT data suggests that while CBCT is a valuable tool, its measurements may lack the absolute reliability required for precise decision-making in cases of pre-existing minimal dentin thickness. For instance, the mean DZ thickness measured with the STD protocol was 1.186 mm. Subtracting the maximum recommended preparation of 0.5 mm would leave a theoretical safety margin that is approximately twice the recommended minimum. While this appears safe on average, the inherent underestimation of CBCT means the true remaining dentin could be even less than calculated, potentially eroding this safety margin in individual cases. A dentin thickness of 0.5 mm measured by CBCT, for example, could correspond to a true thickness of approximately 0.46–0.48 mm (depending on the protocol), a difference that, while numerically small, represents a reduction of 5–9% of the available dentin. In teeth with naturally thin DZ approaching the 0.3 mm threshold, this discrepancy could be the difference between a safe procedure and a perforation. This concern is amplified by micro-CT evidence demonstrating that instrumentation systems can induce microcracks in roots with already reduced dentin thickness [23]. Therefore, while the absolute differences are modest, their clinical impact depends on the baseline dentin thickness and the specific clinical scenario. A cautious interpretation of CBCT-based measurements is imperative, reinforcing the need for conservative preparation strategies in these vulnerable areas.

A paired *t*-test and Bland-Altman analysis were employed to evaluate the agreement between CBCT and micro-CT, following established methodologies [1, 5, 24]. Unlike correlation, which is often misapplied in method-comparison studies [25, 26], Bland-Altman analysis assesses interchangeability by defining the limits of agreement. Our analysis demonstrated that the methods are not interchangeable for submillimetric measurements. Although data points fell within the 95% confidence limits, the mean difference was significantly different from zero, revealing a systematic bias rather than a complete lack of agreement. This finding indicates that CBCT consistently underestimates dentin thickness relative to micro-CT, with the bias varying by protocol (ranging from 4.5 to 8.6%). While the presence of data points within the limits might suggest some level of agreement, the consistent deviation of the mean difference from zero confirms that CBCT and micro-CT are not metrically equivalent. Importantly, this systematic bias is predictable and, if properly accounted for, may still allow CBCT to serve as a useful clinical tool. The critical distinction is that the relationship between the methods is characterized by limited agreement due to a fixed bias, rather than by random error or interchangeability.

The Bland-Altman analysis is particularly sensitive when measuring structures on a submillimeter scale. As noted in the literature, measurements approaching zero can compress the apparent limits of agreement, potentially creating a bias that masks the true clinical discrepancy [24]. Crucially, when the confidence limits of the agreement are wider than the structure being measured, the imaging protocol lacks the necessary resolution for accurate assessment. This principle directly applies to our investigation of the DZ. The fact that even the highest-resolution protocol (HIRE, 0.125 mm voxel size) demonstrated a consistent underestimation confirms that current CBCT spatial resolution remains a fundamental limitation for reliably measuring these delicate anatomical structures.

Regarding the measurement of root dentin thickness, the examiner showed excellent intra-examiner agreement, with a mean ICC value of 0.92. This data demonstrates the examiner’s reliability, ensuring the accuracy of the measurement results in this work. Studies that also compared CBCT technology with micro-CT found ICCs greater than 0.90, similar to the present study [5, 6, 8].

All CBCT protocols in this study yielded measurements significantly different from the µCT gold standard, confirming a fundamental limitation for submillimetric assessment. While protocol parameters like voxel size, FOV, and radiation dose collectively influence diagnostic accuracy [12], our results demonstrate that voxel size alone is insufficient to ensure accuracy. This is consistent with the broader limitations of CBCT technology. For instance, Tolentino et al. [1] reported that even a 0.08 mm voxel size failed to detect apical isthmuses, attributing this to detector limitations and the partial volume effect. This phenomenon, where a single voxel averages the densities of different materials, becomes critical when the voxel size is larger than the fine detail being measured [1, 27], such as the thin dentin walls of the DZ. Our findings align with this principle. The HIRE protocol (0.125 mm voxel) showed the least underestimation, yet a significant discrepancy remained. Furthermore, larger voxel sizes (HIFI, STD, i-CAT) did not cause a proportional loss of structural visualizability. This pattern indicates that inherent imaging artifacts – noise and the partial volume effect – have a greater impact on measurement accuracy than voxel size in isolation [19, 28]. This is compounded by FOV selection; larger FOVs (e.g. i-CAT) often sacrifice resolution, while limited FOVs (e.g. Accuitomo 170) optimize it for localized areas [26]. Consequently, even high-resolution CBCT protocols face spatial resolution challenges that limit reliable measurement of delicate structures like the DZ.

Since CBCT remains an essential clinical tool, its measurements must be interpreted with caution in critical anatomical regions like the DZ. The use of CBCT for submillimetric measurements should account for the possibility of underestimation, and clinical decisions should be based not only on these values but also on the clinician’s expertise and complementary assessments. This concern is supported by recent CBCT-based anatomical studies, which emphasize the importance of evaluating dentin thickness carefully in the DZ to prevent complications such as perforations and [16]. This anatomical vulnerability is not exclusive to mandibular molars; studies on maxillary molars have also shown reduced dentin thickness in regions such as the MB2 canal, emphasizing the need for caution during instrumentation across different molar types [29].

Anatomical studies have demonstrated considerable variability in the vertical position of the DZ, with minimum dentin thickness often occurring in the middle third of the root at levels ranging from 2 to 6 mm apical to the furcation, depending on individual root morphology, groove depth, and tooth length [18]. Nevertheless, the 2 mm level was adopted in the present study for several reasons. This measurement level represents a reproducible anatomical landmark that can be reliably identified on both CBCT and micro-CT images, as the furcation provides a consistent radiographic reference point. Moreover, this level has been widely employed in previous CBCT versus micro-CT comparison studies, allowing direct comparability of our findings with the existing literature [18, 30]. Furthermore, the 2 mm level corresponds to the coronal aspect of the middle third of the root, a region of particular clinical relevance, as it is frequently accessed during mechanical instrumentation and represents the area where iatrogenic complications such as perforation are most likely to occur. While we acknowledge that the true DZ may be located at different levels in individual teeth, the use of a single standardized measurement point was essential to ensure methodological consistency across protocols and to enable meaningful statistical comparisons. Future investigations incorporating multi-level assessments (e.g. 2, 4, and 6 mm) would further elucidate the three-dimensional distribution of dentin thickness in the DZ and could inform more individualized clinical decision-making.

Some limitations should be acknowledged, although several are inherent to the study design. The ex vivo nature of this investigation, while essential for establishing micro-CT as the gold standard reference, may not fully replicate the clinical environment due to factors such as patient movement and the presence of surrounding anatomical structures. The sample was restricted to mandibular molars, which limits generalizability to other tooth groups, though this focus allowed for detailed, standardized assessment of a clinically relevant and anatomically challenging region [29]. Similarly, the evaluation of only two CBCT systems (Accuitomo 170 and i-CAT Classic) reflects a pragmatic choice, as these represent commonly used devices with distinct detector technologies, yet our findings may not extend to other manufacturers [5]. Measurements were performed by a single trained examiner, precluding interobserver reliability assessment; however, the high intraobserver agreement (ICC = 0.92) supports measurement consistency. The analysis was restricted to a single measurement level (2 mm below the bifurcation), a methodological choice adopted from established protocols to enable comparability with prior studies [1, 18], though the DZ may extend along multiple root levels. Although soft tissue simulators helped approximate clinical conditions, limitations include the absence of metallic restorative materials (which may degrade CBCT image quality) and the lack of formal quantitative registration error assessment, though manual registration was visually inspected in all orthogonal planes. [1, 5, 31]. Despite these limitations, the systematic and consistent nature of the observed measurement bias across protocols strengthens the validity of our findings and supports the clinical recommendations provided.

## Conclusion

Under the specific experimental conditions of this study, all CBCT protocols significantly underestimated dentin thickness in the DZ compared to the micro-CT reference standard, with discrepancies ranging from 4.5 to 8.6%. While the HIRE protocol (0.125 mm voxel) demonstrated the smallest discrepancy, no CBCT protocol achieved metric equivalence to micro-CT for these submillimetric measurements. Therefore, within the scope of this investigation, caution is recommended when interpreting dentin thickness values obtained by CBCT for preoperative assessment of the DZ in mandibular molars, as even small differences could impact the safety of endodontic instrumentation.

## Data Availability

Data available on request from the authors.
